# Bone-turnover biomarkers as potential prognostic factors in sudden sensorineural hearing loss: A prospective cohort study

**DOI:** 10.3389/fneur.2022.980150

**Published:** 2022-08-25

**Authors:** Xiaoyan Chen, Zhong Zheng, Lili Xiao, Chengqi Liu, Ying Shen, Ning Ma, Hongjun Dong, Shankai Yin, Yanmei Feng

**Affiliations:** ^1^Department of Otolaryngology-Head and Neck Surgery, Otolaryngology Institute, Shanghai Jiao Tong University Affiliated Sixth People's Hospital, Shanghai, China; ^2^Shanghai Key Laboratory of Sleep Disordered Breathing, Department of Otolaryngology-Head and Neck Surgery, Shanghai Jiao Tong University Affiliated Sixth People's Hospital, Shanghai, China; ^3^Department of Otorhinolaryngology, Zhangjiagang TCM Hospital Affiliated to Nanjing University of Chinese Medicine, Suzhou, China

**Keywords:** sudden sensorineural hearing loss, bone-turnover biomarkers, osteoporosis, prognosis, β-carboxy terminal crosslinked telopeptide of type 1 collagen, N-terminal-midfragment of osteocalcin

## Abstract

**Objectives:**

This study aims to explore the relationship between bone-turnover biomarkers and the recovery of SSNHL to provide clues for further improvements in etiological research and predictors.

**Methods:**

The medical history, hearing thresholds, biomarkers of bone-turnover, and related hormones of 117 SSNHL patients were collected prospectively between August 2018 and December 2021. Linear correlation and logistic regression models were applied to examine the association between bone-turnover biomarkers and the prognosis of SSNHL.

**Results:**

Age, the incidence of vertigo, pure tone average of the impaired frequencies (PTA_impairedfre_), and the levels of bone turnover [including alkaline phosphatase (ALP), β-carboxy terminal crosslinked telopeptide of type 1 collagen (β-CTX), and N-terminal-midfragment of osteocalcin (N-MID)] were higher in the nonresponders than responders (*P* < 0.05). Logistic regression showed that the age (OR = 1.035, *P* = 0.027), time to treatment (OR = 1.157, *P* = 0.038), PTA_impairedfre_ (OR = 1.031, *P* = 0.008), and β-CTX (OR = 1.004, *P* = 0.001) were independent risk factors for the prognosis of SSNHL. In the women SSNHL subgroup, age, postmenopause percentage, PTA_impairedfre_, the activity of ALP, levels of β-CTX, and N-MID were significantly higher in the nonresponders than the responders (*P* < 0.05). Compared to the men SSNHL subgroup, β-CTX has a higher correlation coefficient and predictive efficiency in the women SSNHL subgroup, and logistic regression showed that β-CTX (OR = 1.004, *P* = 0.004) was an independent risk factor for the women SSNHL.

**Conclusions:**

Bone-turnover biomarkers are risk factors for poor prognosis in SSNHL, especially β-CTX. The differences were significant in women SSNHL, which may be related to the rapid regression of estrogen after menopause that leads to the occurrence of osteoporosis with a high conversion rate.

## Introduction

Sudden sensorineural hearing loss (SSNHL) is a common otology emergency defined as sudden hearing impairment of no <30 dB over at least three consecutive frequencies on the audiogram within 3 days and with unclear predisposing factors (1). Since its etiology is multifaceted, patient response to treatment varies (2, 3). Detailed examination to identify risk factors and receive timely treatment is thus very important to improve hearing loss of SSNHL. Currently, there is a lack of accurate prognostic indicators, but studies have suggested elements such as metabolism, blood coagulation, inflammation, immunity, and oxidative stress are potential factors (2, 4, 5). There have also been reports on the association between osteoporosis and the occurrence of SSNHL. Yeh et al. (6) suggested that the incidence of SSNHL in patients with osteoporosis was 1.76 times (95% CI: 1.33–2.34, *P* < 0.001) higher than that in patients without osteoporosis. Kim et al. (7) tracked and analyzed 68,241 osteoporosis patients aged ≥50 years old. They found that this group was at a higher risk of SSNHL (adjusted HR: 1.56, 95% CI: 1.37–1.78, *P* < 0.001), and women osteoporosis patients aged ≥60 had a higher incidence of SSNHL (aged 60–69 years, adjusted HR: 1.67, 95% CI: 1.34–2.08, *P* < 0.001; aged ≥ 70 years, adjusted HR: 1.90, 95% CI: 1.29–2.79, *P* < 0.001), which may be related to rapid estrogen withdrawal after menopausal.

The contributions of osteoporosis toward SSNHL could be attributed to the followings: (1) Demineralization of the petrosal or cochlear bone which is associated with hearing loss in the elderly, Paget disease, and otosclerosis. An abnormal increase in bone resorption leads to high calcium concentrations in the endolymph thus interfering with endolymph potential (8, 9). Such interference may disrupt the desirable low calcium concentration in the endolymph that is critical for the homeostasis of endolymphatic potential and sensory transduction in cochlear hair cells (10). (2) Decreased bone mineral density (BMD) results in multiple microfractures of the ossicles. Secondary bone remodeling may also alter the normal sound transmission characteristics of the middle ear, showing a decrease in the middle ear resonance frequency, and an increase in static compliance (11). (3) Osteoprotegerin acts as a key regulator of bone remodeling. The temporal bone in osteoprotegerin knockout mice exhibits abnormal bone remodeling. Osteoprotegerin deficiency causes demyelination and degeneration of the cochlear nerve *in vivo*, and also activates extracellular regulated protein kinases, sensitizing spiral ganglion cells to apoptosis (12). Although there is evidence that osteoporosis contributes to the increased incidence of SSNHL, the relationship between the bone-turnover biomarkers with the prognosis of SSNHL is still unclear. Bone-turnover biomarkers [including alkaline phosphatase (ALP), β-carboxy terminal crosslinked telopeptide of type 1 collagen (β-CTX), N-terminal midfragment of osteocalcin (N-MID)] and related biomarkers [including serum calcium, serum phosphorus (P), 25 hydroxyvitamin D (25(OH)D), parathyroid hormone (PTH), and calcitonin] can be used as a risk assessment tool for post-menopausal osteoporosis, and some studies even suggested that β-CTX to be better than BMD in predicting senile fractures (13, 14). This study prospectively analyzed the correlation between bone-turnover biomarkers and hearing recovery in SSNHL patients. Our findings could provide clues for further understanding the pathogenesis and improving the treatment effect of SSNHL.

## Materials and methods

### Study population

A total of 117 patients who were first diagnosed with SSNHL and met the inclusion criteria between August 2018 and December 2021 were prospectively enrolled. To investigate the potential relationship between bone-turnover biomarkers and SSNHL at all frequencies, we included SSNHL patients with a decrease in hearing ≥ 30 dB affecting at least 3 consecutive frequencies on the window within 72 h and with unclear predisposing factors (1). The research process fully followed the Declaration of Helsinki. The research protocol was approved by the Ethics Committee of the Sixth People's Hospital Affiliated to Shanghai Jiaotong University [2018-KY-036(K), 2018.07.24] and informed consent was obtained from all participants. Inclusion criteria: (1) Met the above diagnostic criteria for SSNHL. (2) Had not received systematic and standardized treatment before presenting to the outpatient clinic. (3) Received treatment within 2 weeks after hearing loss at our department. (4) Patients adhered to treatment and follow-up. Exclusion criteria: (1) Suffered from migraine or ear diseases that interfere with the diagnosis of SSNHL, such as chronic otitis media, acoustic trauma or ear surgery, conductive hearing loss, and Meniere's disease. (2) Suffered from diabetes, thyroid disease, liver and renal function disfunction, bone fracture, severe organ dysfunction, and malignancy that significantly affect bone turnover and metabolism. (3) Lost to follow-up or incomplete data. (4) <18 years old. (5) Took drugs that interfered with bone turnover and metabolism.

### Data collection

A detailed medical history was obtained from all patients, including baseline characteristics (age, sex, BMI, comorbidities) and clinical characteristics (affected side, accompanying symptoms, time to treatment, hearing loss levels after onset and treatment). All hearing assessments were performed in standard shielding rooms, and pure tone audiometry was performed for both air and bone conduction at 0.125, 0.25, 0.5, 1, 2, 4, and 8 kHz with appropriate masking before and after 1 month of systemic treatment. Pure tone average of the impaired frequencies (PTA_impairedfre_) were calculated by the average hearing thresholds of the impaired frequencies (0.125–8 kHZ) after onset, while the hearing threshold shift is calculated using initial PTA_impairedfre_ minus the PTA_impairedfre_ after treatment. Computed tomography of the temporal bone or magnetic resonance imaging of the inner ear was performed in all patients to rule out ear structural abnormalities and tumors. Considering the degree of patient cooperation, qCT and BMD examinations have not been performed. Before glucocorticoid therapy, blood samples were obtained from the antecubital veins between 6 and 7 a.m. after an overnight fast. 4°C low-temperature centrifugation (3,000 r/min, centrifugal radius 10 cm) was then performed to separate the upper serum. Bone-turnover and related biomarkers were assayed using an automatic biochemical analyzer (Roche cobas 8,000 e602, Switzerland; hitachi labospect 008 as, Japan). Serum calcium exists in the form of ionized calcium and bound calcium, and most of the bound calcium is carried by serum albumin. The clinical test of serum calcium is greatly affected by serum albumin, thus serum albumin-adjusted calcium (S-Ca) was used. S-Ca (mmol/l) = Serum total calcium (mmol/l)-0.025 × Serum albumin concentration (g/L) + 1.0 (mmol/L) (15).

### Treatment and evaluation

According to the USA guidelines of the SSNHL (16), all patients received a 7–14 days course of intravenous glucocorticoids (prednisone 1 mg/kg/day for 3–5 days, maximum dosage ≤60 mg, followed by a reduced dosage for the remaining days according to the hearing improvement). Tympanic injection and/or hyperbaric oxygen therapy were considered in patients with refractory SSNHL. Patients were divided into two groups based on the hearing recovery that was observed within 1 month of follow-up: responders (the hearing threshold shift ≥15 dB, or restored to normal/unaffected contralateral hearing thresholds) and nonresponders (the hearing threshold shift <15 dB) (16).

### Statistical analysis

Statistical analysis was performed using SPSS 26.0, GraphPad Prism 8.0 and R 4.0.4 software. Descriptive variables were expressed as mean ± standard deviation, median (interquartile range), or percentage. Independent samples *t*-test was used to compare the average values of the variable with normal distribution. Mann-Whitney *U-*test was used to compare the medians of the continuous variables that do not satisfy the normal distribution. The Chi-square test was used to compare the categorical variables. Spearman correlation analysis was performed to assess the association between biochemical markers of bone turnover and threshold shift. The backward conditional logistic regression was used to estimate the odds ratios (OR) and 95% confidence intervals (CI) for the correlation between bone-turnover biomarkers and therapeutic effect. The collinearity of all continuous variables was examined before performing the logistic regression using the variance inflation factor. To detect the most significant parameter related to the outcome of SSNHL and to determine a cut-off value, the receiver operating characteristic (ROC) curve analysis test was used. *P* < 0.05 was considered significant for all tests.

## Results

### Baseline and clinical characteristics of participants

**Figure 1:**
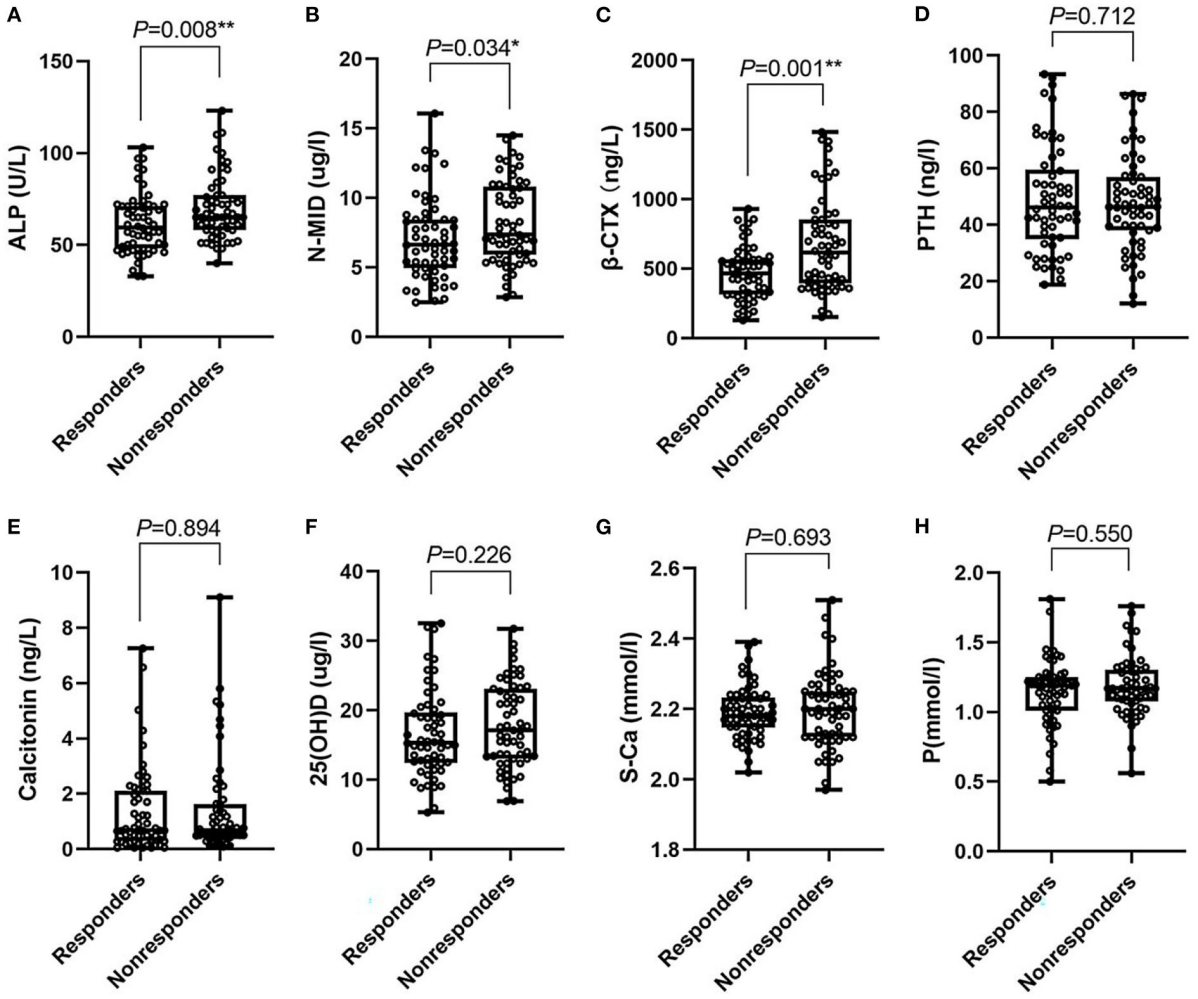
Box-plot. **(A–H)** Represent the median (min-max) of ALP, N-MID, β-CTX, PTH, calcitonin, 25(OH)D, S-Ca, P in responders and nonresponders, respectively. ALP, Alkaline phosphatase; N-MID, N-terminal-midfragment of osteocalcin; β-CTX, β-carboxy terminal crosslinked telopeptide of type 1 collagen; PTH, parathyroid hormone; 25(OH)D, 25 hydroxyvitamin D; S-Ca, serum albumin-adjusted calcium; P, serum phosphate. *0.01 ≤ *P* < 0.05; ** 0.001 ≤ *P* < 0.01.

The baseline characteristics, clinical characteristics, bone-turnover, and related biomarkers of the subjects are detailed in Table 1 and Figure 1. The median age in the treatment nonresponders was 54.00 higher than that in the responders, which was 38.00 (*P* < 0.05). Gender, hypertension, and BMI levels between the two groups yielded no difference. Compared to the responders, the incidence of vertigo, PTA_impairedfre_, the activity of ALP, the expression levels of N-MID, and β-CTX were higher in the nonresponders (*P* < 0.05). However, no statistical differences were found for S-Ca, P, 25(OH)D, PTH, and calcitonin between the two groups.

**Table 1:** Characteristics of data in SSNHL patients.

	**Responders (*n* = 58)**	**Nonresponders (*n* = 59)**	***P* value**
**Baseline characteristics**
Age (years)^a^	38.00 (32.00–55.00)	54.00 (38.00–63.00)	0.005*
Gender (men, %)^b^	21 (36.21%)	30 (50.85%)	0.110
hypertension (%)^b^	7 (12.07%)	10 (16.95%)	0.454
BMI (kg/m2)^a^	23.53 (21.23–25.39)	22.69 (21.20–25.42)	0.719
**Clinical characteristics**
Affected side (left, %)^b^	37 (63.79%)	33 (55.93%)	0.386
Tinnitus (%)^b^	33 (56.90%)	36 (61.02%)	0.651
Vertigo (%)^b^	12 (20.69%)	24 (40.68%)	0.019*
Ear fullness (%)^b^	11 (18.97%)	15 (25.42%)	0.401
Time to treatment (days)^a^	4.00 (2.00–7.00)	4.00 (2.00–7.00)	0.306
PTA_impairedfre_ (dBHL)^a^	67.93 (44.47–82.32)	85.71 (75.00–97.86)	0.000*
Threshold shift (dB)^a^	29.65 (21.07–41.43)	1.43 (0.00–6.43)	0.000*
**Laboratory variables**
ALP (U/L)^a^	59.50 (48.00–71.00)	65.00 (58.00–77.00)	0.008*
N-MID (ug/l)^a^	6.62 (4.93–8.44)	7.35 (5.91–10.80)	0.034*
β-CTX (ng/l)^a^	465.85 (313.80–567.08)	617.40 (394.70–853.80)	0.001*
PTH (ng/l)^c^	48.91 ± 19.01	47.68 ± 16.84	0.712
Calcitonin (ng/L)^a^	0.69 (0.29–2.10)	0.69 (0.46–1.63)	0.894
25(OH)D (ug/l)^a^	15.31 (12.41–19.70)	17.10 (13.03–23.07)	0.226
S-Ca (mmol/l)^a^	2.18 (2.14–2.23)	2.20 (2.12–2.26)	0.693
P(mmol/l)^a^	1.17 (1.08–1.30)	1.19 (1.01–1.25)	0.550

a Values are given as median with its interquartile range (25–75th) in parentheses.

b Values are given as the number of cases and the percentage in parentheses.

c Values are given as mean ± standard deviation.

* P < 0.05.

BMI, Body mass index; PTA_impairedfre_, Pure tone average of the impaired frequencies (0.125–8kHZ); ALP, Alkaline phosphatase; N-MID, N-terminal-midfragment of osteocalcin; β-CTX, β-carboxy terminal crosslinked telopeptide of type 1 collagen; PTH, parathyroid hormone; 25(OH)D, 25 hydroxyvitamin D; S-Ca, Serum albumin-adjusted calcium; P, Serum phosphorus.

### Binary logistic regression model

**Figure 2:**
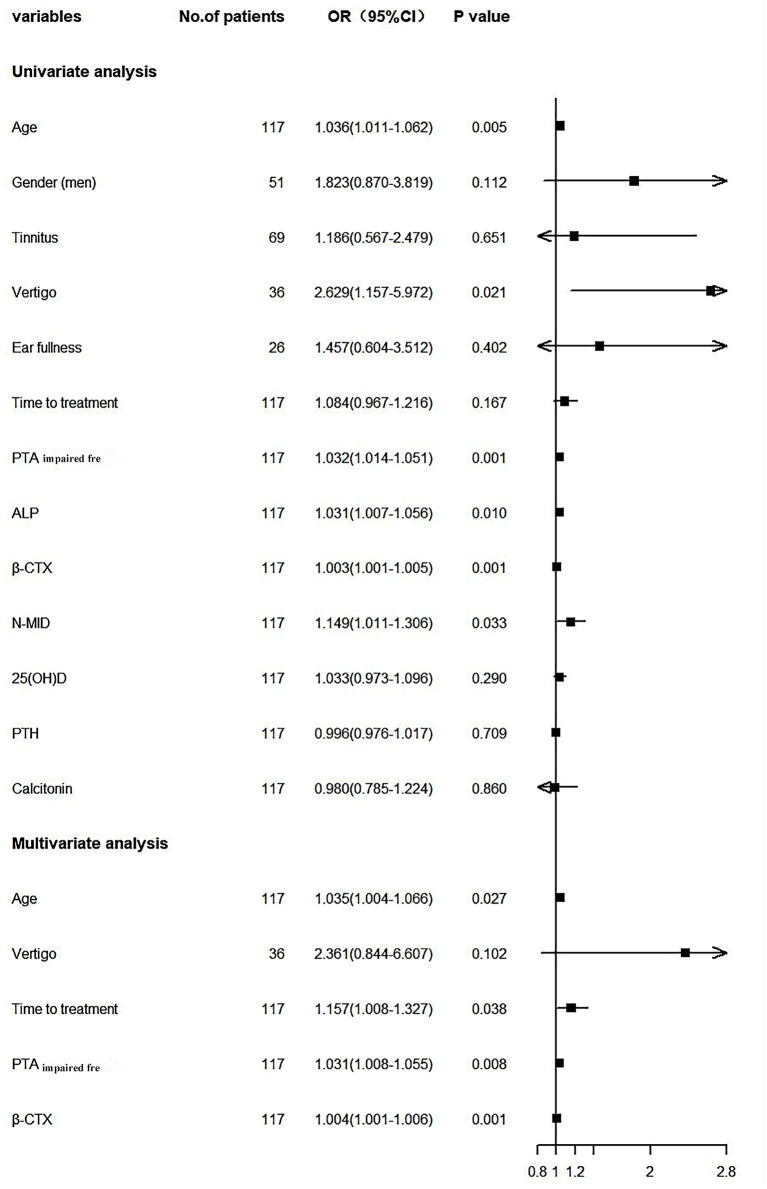
Forest plot. PTA_impairedfre_, Pure tone average of the impaired frequencies (0.125–8 kHZ); ALP, Alkaline phosphatase; β-CTX, β-carboxy terminal crosslinked telopeptide of type 1 collagen; N-MID, N-terminal-midfragment of osteocalcin; 25(OH)D, 25 hydroxyvitamin D; PTH, parathyroid hormone.

We incorporated significant influential factors into the regression model (Figure 2), including age, gender, tinnitus, vertigo, ear fullness, time to treatment, PTA_impairedfre_, bone-turnover biomarkers, and related hormones. And univariate logistic regression showed that age, vertigo, PTA_impairedfre_, ALP, N-MID, and β-CTX were risk factors affecting the prognosis of SSNHL. The above indicators that were statistically significant were included in the multivariate logistic regression model along with the time to treatment. Age, time to treatment, PTA_impairedfre_, and β-CTX were found to be the independent risk factors for the prognosis of SSNHL after adjusting for confounders. The C-index was 0.80 (95% CI: 0.72–0.88, *P* = 1.69e-05), and the chi-square of Hosmer and Lemeshow Test was 4.28 (*P* = 0.83), indicating that the predictive ability of the model was acceptable.

### The characteristics of SSNHL in men and women subgroups

Bone-turnover biomarkers showed different growth trends in different developmental stages of men and women patients. Bone-turnover biomarkers increase rapidly and significantly during the menopausal transition and then the concentrations remain high and stable, which is higher than men at the same age (17, 18). Grouping by gender, we found that age [41.00 (32.50–53.50) vs. 54.00 (45.50–59.50), *P* = 0.011], postmenopause percentage [11 (29.73%) vs. 19 (65.52%), *P* = 0.004], PTA_impairedfre_ [66.43 (35.84–84.65) vs. 89.29 (51.07–97.86), *P* = 0.007], the activity of ALP [58.00 (47.50–71.00) vs. 65.00 (58.50–72.50), *P* = 0.033], the levels of N-MID [6.37 (4.81–8.44) vs. 7.35 (6.26–11.04), *P* = 0.045], and β-CTX [443.40 (311.85–555.50) vs 627.00 (430.90–839.80), *P* = 0.002] were significantly lower in the responders than the nonresponders of the women SSNHL subgroup. However, the age [37.00 (31.50–57.50) vs. 52.50 (32.75–65.25), *P* = 0.143], the activity of ALP [63.00 (49.00–73.00) vs. 65.50 (57.75–83.75), *P* = 0.148], the expression of N-MID [6.64 (5.40–9.32) vs. 7.28 (5.53–10.75), *P* = 0.479], and β-CTX [514.30 (343.30–610.70) vs. 543.40 (372.10–976.03), *P* = 0.141] were missing statistical significance in the men subgroup.

### Binary logistic regression model in men and women SSNHL subgroups

**Figure 3:**
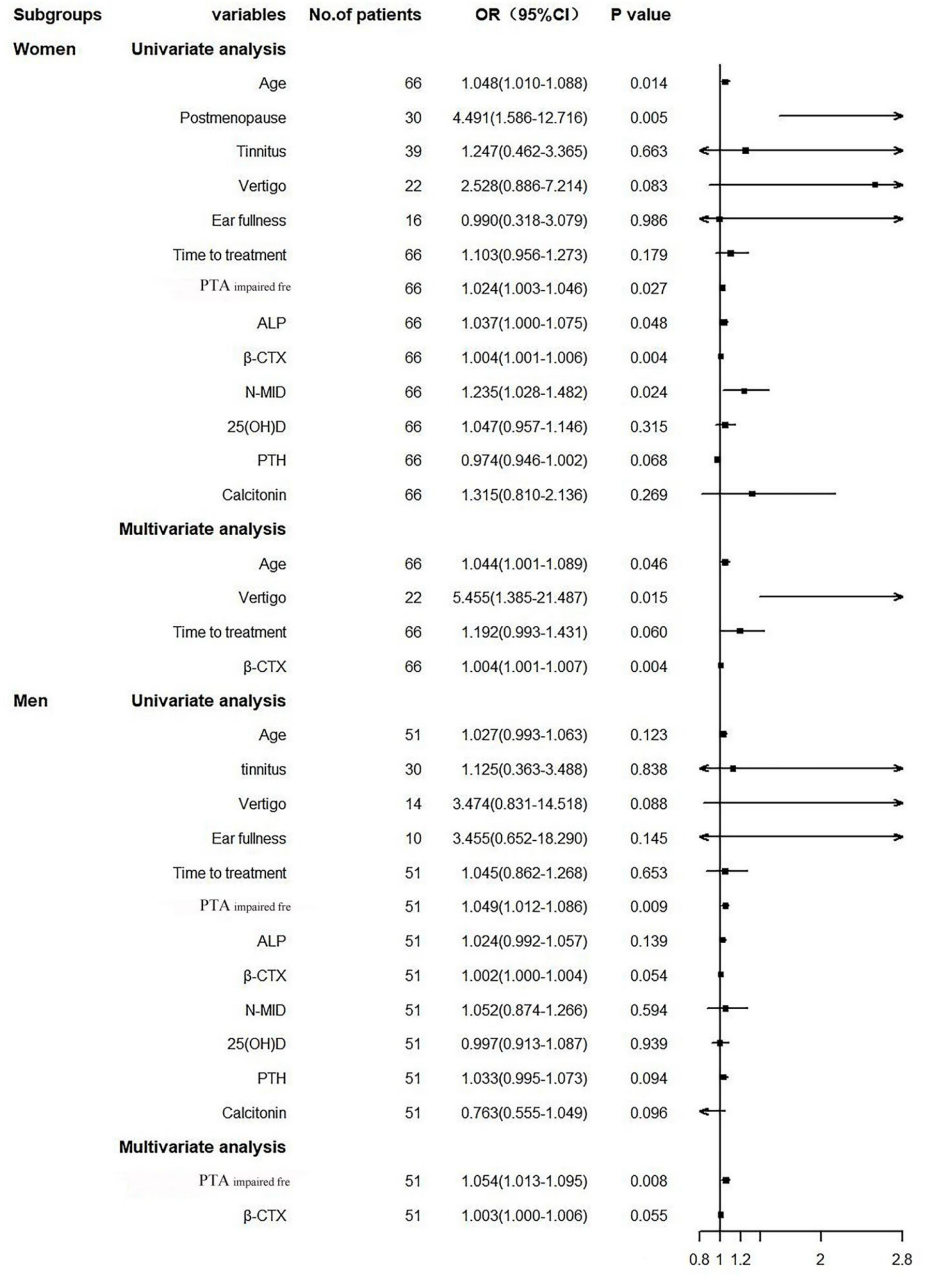
Forest plot. PTA_impairedfre_, Pure tone average of the impaired frequencies (0.125–8 kHZ); ALP, Alkaline phosphatase; β-CTX, β-carboxy terminal crosslinked telopeptide of type 1 collagen; N-MID, N-terminal-midfragment of osteocalcin; 25(OH)D, 25 hydroxyvitamin D; PTH, parathyroid hormone.

The univariate logistic regression model showed that age, postmenopause percentage, ALP, N-MID, and β-CTX were risk factors affecting the prognosis of women SSNHL, but not in the men SSNHL subgroup (Figure 3). Other than the variable of postmenopause, we included the same variables in Figure 2 to construct the multivariate logistic regression model, and found that β-CTX was an independent risk factor for the women SSNH subgroup, as it was more statistically significant than the men SSNHL subgroup (Figure 3).

### ROC curves

**Figure 4:**
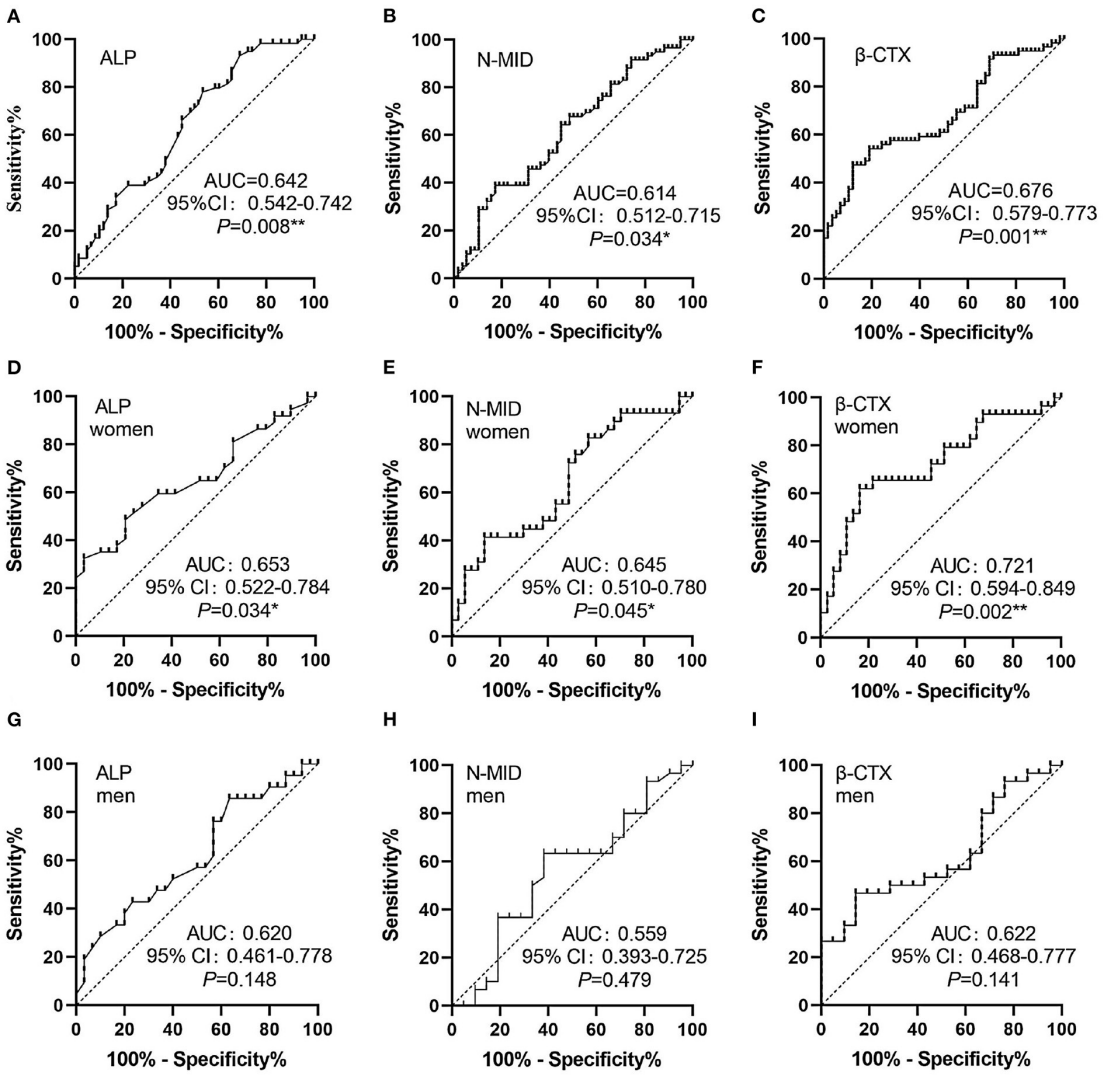
ROC curves. **(A–C)** ROC curves of ALP, N-MID and β-CTX, respectively. **(D–F)** ROC curves of ALP, N-MID and β-CTX in women SSNHL, respectively. **(G–I)** ROC curves of ALP, N-MID and β-CTX in men SSNHL, respectively. *0.01 ≤ *P* < 0.05, **0.001 ≤ *P* < 0.01. AUC, areas under the curves; ALP, Alkaline phosphatase; N-MID, N-terminal-midfragment of osteocalcin; β-CTX, β-carboxy terminal crosslinked telopeptide of type 1 collage.

ROC curves analysis was performed to determine whether bone-turnover biomarkers could predict the outcome of SSNHL (Figure 4). ALP, N-MID, and β-CTX were found to be incomplete predictors of SSNHL (Figures 4A–C). It can be seen that β-CTX has the best predictive performance, especially in the women SSNHL subgroup (Figure 4F).

### Linear correlation analysis

**Figure 5:**
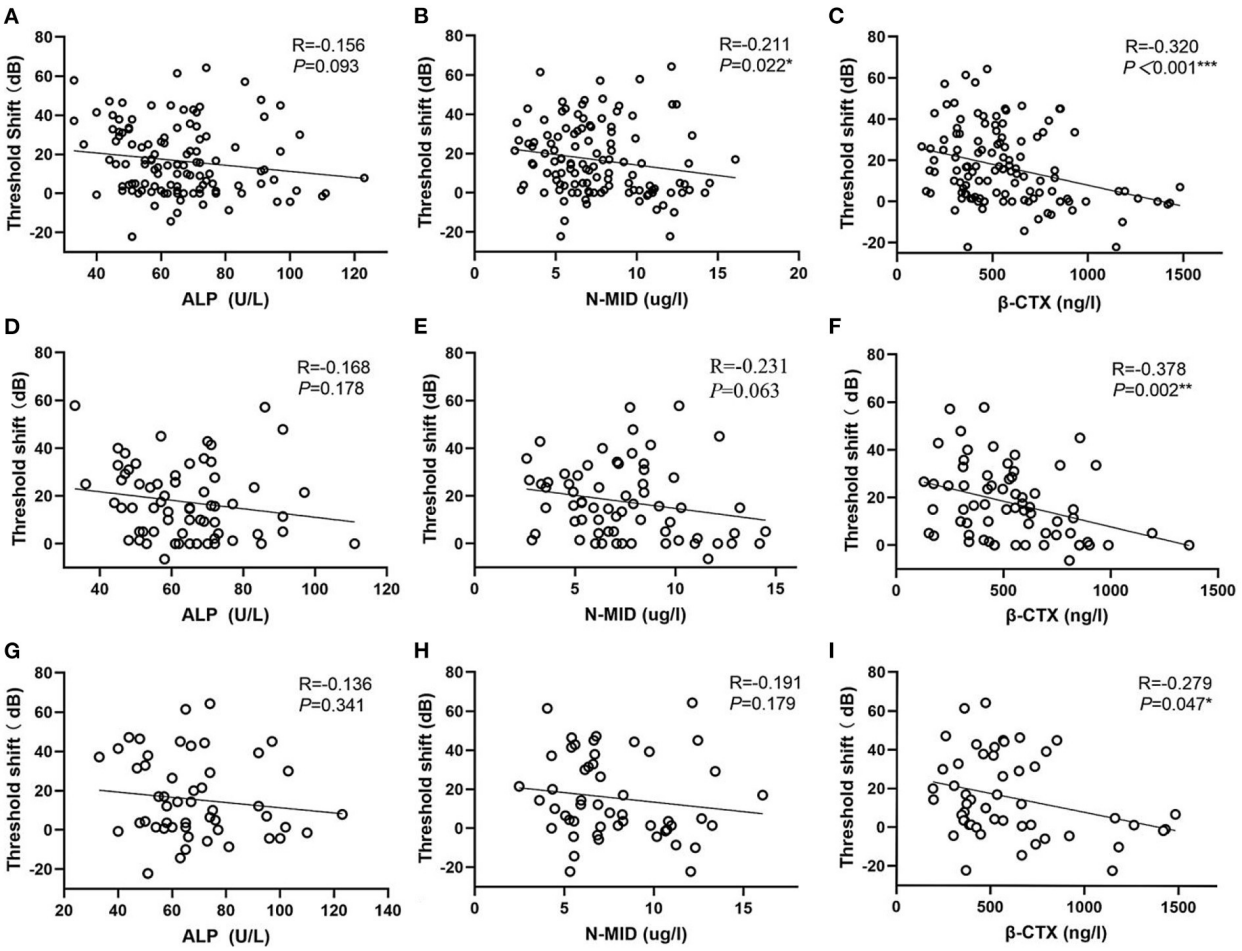
Scatter plots. **(A–C)** Represent the relationship of ALP, N-MID and β-CTX vs. the threshold shift, respectively. **(D–F)** Represent the relationship of ALP, N-MID and β-CTX vs. the threshold shift in women SSNHL, respectively. **(G–I)** Represent the relationship of ALP, N-MID and β-CTX vs. the threshold shift in men SSNHL, respectively. *0.01 ≤ *P* < 0.05, **0.001 ≤ *P* < 0.01, ***P < 0.001. ALP, Alkaline phosphatase; N-MID, N-terminal-midfragment of osteocalcin; β-CTX, β-carboxy terminal crosslinked telopeptide of type 1 collage.

Among the bone-turnover biomarkers, only ALP, N-MID, and β-CTX had statistical differences between the responders and nonresponders. Therefore, we performed a linear correlation analysis (Figure 5) and found that N-MID and β-CTX were negatively and linearly correlated to the threshold shift of SSNHL (Figures 5B,C). In particular, β-CTX was negatively and linearly correlated with the threshold shift in both men and women SSNHL subgroups (Figures 5F,I).

### Characteristics of pre-menopausal and post-menopausal SSNHL subgroups

Bone-turnover biomarkers increased with rapid regression of estrogen after menopause in women, which leads to osteoporosis with a high conversion rate (19). So we speculated that post-menopausal women have a poor prognosis of SSNHL. Compared with pre-menopausal women, post-menopausal women with SSNHL has a lower treatment effective rate (11/30 vs. 26/36, *P* = 0.004), lower threshold shift of recovery [9.50 (0.00–28.75) vs. 16.91 (9.47–26.43), *P* = 0.101], a higher PTA_impairedfre_ [81.79 (58.75–97.86) vs. 66.86 (35.00–89.64), *P* = 0.026], a higher activity of ALP [69.50 (61.00–74.00) vs. 57.00 (48.00–68.50), *P* = 0.002], a higher expression levels of β-CTX [606.80 (439.78–833.05) vs. 423.00 (311.48–587.40), *P* = 0.004]. It can be seen that the occurrence of high conversion rate osteoporosis after menopausal may be one of the reasons for the poor prognosis of SSNHL.

## Discussion

Previous studies have suggested that vertigo, severe hearing loss, and delayed time to treatment (>15 days) were risk factors for the prognosis of SSNHL, but only severe hearing loss and delayed time to treatment were independent risks factor. In addition, the recovery rate was low in patients >60 years old (20). We found that age, vertigo, and PTA_impairedfre_ were statistically different between the responders and nonresponders. Multivariate logistic regression supported the age, PTA_impairedfre_, and time to treatment as independent prognostic risk factors for SSNHL, consistent with clinical consensuses.

ALP, N-MID, and β-CTX are markers of bone formation and resorption, which can be easily detected and directly reflect the change in bone metabolism before bone morphological change (14, 21). β-CTX is a C-terminal cross-linked peptide of type I collagen, which is produced in the process of collagen decomposition and can be used to indirectly reflect the function and activity of osteoclasts in the skeletal system (22). β-CTX was stable for 8 h at room temperature and 48 h at 4°C in serum (23). It could be considered an ideal and stable marker to reflect bone resorption (22). N-MID is the hydrolytic fragment of osteocalcin secreted by osteoblasts and can be used to reflect the process of bone formation. The declining N-MID and β-CTX were significantly associated with a short-term positive change in Lumber spine 1-4 BMD of postmenopausal women over 1 year (14). ALP is a group of glycoproteases that can hydrolyze phosphates under alkaline conditions. One study found that 42 individuals with persistently low serum ALP have an overall reduction of bone turnover, even in the absence of overt manifestations of low BMD (24). We found that ALP, β-CTX, and N-MID were statistically different between the responders and nonresponders. Correlation analysis found that β-CTX and N-MID have a negative linear correlation with the recovery of hearing loss, and a logistic regression model was established revealing that β-CTX could be used as an independent risk factor for prognosis. For the first time, we found that β-CTX is a prognostic indicator of SSNHL, which provides clues for further improving the etiological research and clinical treatment of SSNHL. Lee et al. found that serum osteocalcin and urinary deoxypyridinoline levels in patients with BPPV were significantly elevated compared with those of controls. Multiple logistic regression analyses showed that osteoporosis and vitamin D deficiency were associated with BPPV (25). The aminoterminal propeptide of type I procollagen (PINP) and β-CTX levels in male patients with BPPV had not statistically significant compared to the control group (26). But a large, single-institution study and subgroup analysis of BMD and serum levels of 25(OH)D and bone turnover markers in postmenopausal female patients with BPPV, and none of these analyses were significantly different between the different types (27). We can speculate that bone metabolism affects the occurrence of BPPV, but not the type. Whether bone metabolism is related to the occurrence of SSNHL or affects other ear diseases needs further study.

Women with osteopenia/osteoporosis had significantly higher average hearing thresholds at all frequencies by pure tone average than women with normal BMD. The multivariate logistic regression model showed that age and lumbar spine BMD were associated with the presence of hearing loss (>25 dB), suggesting that the presence of reduced BMD in post-menopausal women may contribute to a higher prevalence of age-related hearing loss (28). Several studies also support that SSNHL is closely related to post-menopausal osteoporosis (6, 7). Timely detection of bone-turnover biomarkers and correction of osteoporosis may be beneficial for the treatment of SSNHL (29–31). We found that the proportion of post-menopausal women and the activity of ALP, the expression levels of β-CTX and N-MID in the nonresponders of women SSNHL were higher with statistically significant differences compared with the responders. And the median of β-CTX in the nonresponders was significantly higher than that in the general population of postmenopausal women [509.84 ng/L (356.00–646.00)] (32). Post-menopausal women with SSNHL had a poor hearing recovery, high hearing loss, and high activity of ALP, high expression levels of β-CTX. It can be speculated that post-menopausal women with rapid estrogen regression contribute to a high conversion rate of osteoporosis, thus leading to a poor prognosis of SSNHL.

25(OH)D, PTH, and calcitonin are representative hormones of bone metabolism. The mutations of 25(OH)D receptor b in zebrafish lead to defects in ear development and a reduction in the number of hair cells (33). The concentration of serum 25(OH)D <20 ng/ml is diagnosed as vitamin D deficiency, and 20 ng/ml < serum 25(OH)D < 30 ng/ml is diagnosed as vitamin D insufficient (34). Szeto et al. (35) analyzed the associations between 25(OH)D, PTH, total calcium, BMD, and hearing loss in 1,123 participants aged ≥70 years and found that 25(OH)D <20 ng/mL was the risk factors of low-frequency hearing loss (OR: 2.02, 95% CI: 1.28–3.19) and voice frequency hearing loss (OR: 1.96, 95% CI: 1.12–3.44), but not PTH and total calcium. Ghazavi et al. (36) found that the average vitamin D level in the SSNHL group was 19.28 ± 9.56 ng/ml, which was significantly lower than that in the control group (25.71 ± 11.21 ng/ml, *P* < 0.001), and believed that adequate vitamin D levels were associated with good prognosis. In postmenopausal women, 25(OH)D levels decrease, and timely estrogen supplementation can promote the deposition of serum calcium into the bones (37). The highest value of 25(OH)D in our data was 32.52 ng/mL, where 96.58% (113/117) of patients were at 25(OH)D <30 ng/mL, and 69.23% (81/117) of patients were at 25 (OH)D <20 ng/mL. However, 78.79% (52/66) of women with SSNHL were at 25(OH)D <20 ng/mL, and we did not find an association between 25(OH)D and prognosis of SSNHL.

The results of this prospective study showed that the poor prognosis of SSNHL is related to the high bone-turnover biomarkers in post-menopausal women. Hormones such as estrogen, progesterone, and aldosterone all demonstrate vital roles in sustaining auditory function through either the maintenance of cochlear neurons, up/down regulation of critical molecules (i.e., IGF-1, BDNF, etc.), or generation of the endocochlear potential. With disease and/or age, hormone expression begins to decline drastically, which ultimately affects cochlear structures and the integrity of cochlear cells (38). Williamson et al. observed the effects of estrogen and progesterone on auditory processing in aging ovariectomized female mice. They found that the estrogen significantly upregulated IGF-1 in the cochlea, helping to promote cell health and survival. But progesterone therapy generated conflicting results by both increasing ABR thresholds and slowing temporal processing deficiencies in ABR GIN amplitude levels associated with aging (39). Our study did not include estrogen and BMD as a routine test index in advance, so the analysis of estrogen and BMD variables was absent. A previous study found that increased plasma serotonin appeared as a biomarker of SSNHL (AUC: 98%) (40). Serotonin which is a molecule that interacts with bone cells and has also been suggested to act as a bone mass regulator (41). These are aspects that need to be further improved in the future.

## Conclusion

ALP, β-CTX, and N-MID were significantly different between the responders and nonresponders, especially in the women SSNHL group. β-CTX and N-MID were negatively and linearly correlated with the prognosis of SSNHL, among which β-CTX was an independent risk factor. β-CTX was more significant in the women SSNHL group, which may be related to the rapid regression of estrogen after menopause to leading the occurrence of osteoporosis with a high conversion rate.

## Data availability statement

The raw data supporting the conclusions of this article will be made available by the authors, without undue reservation.

## Ethics statement

The studies involving human participants were reviewed and approved by the Sixth People's Hospital Affiliated to Shanghai Jiao Tong University [2018-KY-036(K), 2018.07.24]. The patients/participants provided their written informed consent to participate in this study.

## Author contributions

XC, ZZ, LX, and CL designed and supervised the research. YS and NM analyzed the data. HD, SY, and YF gave suggestions on the data acquisition and analysis. XC and ZZ wrote the manuscript. LX, CL, YS, and NM participated in manuscript editing. All authors reviewed the manuscript. All authors contributed to the article and approved the submitted version.

## Funding

This study was supported by the National Natural Science Foundation of China (81771015), the First Grant (2020YFC2005201) of the Chinese National Key Research and Development Program (2020YFC2005200), the Shanghai Municipal Commission of Science and Technology (Grant No. 18DZ2260200), and the International Cooperation and Exchange of the National Natural Science Foundation of China (NSFC; 81720108010).

## Conflict of interest

The authors declare that the research was conducted in the absence of any commercial or financial relationships that could be construed as a potential conflict of interest.

## Publisher's note

All claims expressed in this article are solely those of the authors and do not necessarily represent those of their affiliated organizations, or those of the publisher, the editors and the reviewers. Any product that may be evaluated in this article, or claim that may be made by its manufacturer, is not guaranteed or endorsed by the publisher.
